# Moral injury in modern warfare: clinical reflections and implications for military psychiatry

**DOI:** 10.3389/fpsyt.2026.1738799

**Published:** 2026-02-20

**Authors:** Dotan Braun

**Affiliations:** Department of Psychiatry, The Jerusalem Mental Health Center, Jerusalem, Israel

**Keywords:** ethical conflict, institutional responsibility, military psychiatry, modern warfare, moral injury

## Abstract

Moral injury has emerged as a significant dimension of psychological suffering among individuals exposed to ethically compromising situations in war. Unlike post-traumatic stress disorder (PTSD), which is primarily organized around fear-based responses to threat, moral injury centers on violations of deeply held moral expectations involving responsibility, agency, and trust. In this clinical reflection, moral injury is approached not as a psychiatric diagnosis, but as a clinical–moral condition arising from difficulty reconciling actions or survival with an internalized moral framework. Drawing on theoretical models and clinical experience in military psychiatry, the manuscript examines how features of modern warfare—including asymmetrical conflict, technological mediation, blurred civilian–combatant boundaries, and fragmented chains of responsibility—shape moral injury and complicate processes of moral repair. These conditions do not create moral injury *de novo*, but alter how moral responsibility is experienced, narrated, and addressed within military systems. The paper explores the implications of moral injury for military psychiatry and military organizations, highlighting the interface between individual moral suffering and institutional responsibility. It raises questions about how military systems recognize, legitimize, or remain ambivalent toward moral injury, particularly when contrasted with the more established status of PTSD as an honorable cost of service. Attention is given to the limits of symptom-focused interventions when confronted with moral suffering grounded in intact values, and to the need for clinical and organizational frameworks that support moral acknowledgment and repair. The manuscript situates moral injury within broader professional and societal contexts, emphasizing its relevance for psychiatry in contemporary armed conflict.

In recent years, military psychiatry has been confronted with a dimension of psychological suffering that extends beyond fear, hyperarousal, or intrusive recollections. As a psychiatrist working with soldiers and combatants exposed to the extreme moral ambiguity of modern warfare, I have witnessed injuries not only to the psyche but to the moral self. These are wounds of conscience—borne by those who acted, failed to act, or merely survived in circumstances that transgressed their deepest ethical beliefs.

The concept of moral injury has been articulated in several influential yet distinct formulations. Shay emphasized the role of betrayal of “what’s right” by legitimate authority in high-stakes contexts, while Litz and colleagues framed moral injury as the lasting psychological, social, and spiritual impact of perpetrating, failing to prevent, or witnessing acts that transgress deeply held moral beliefs (1–3). More recent integrative models have highlighted the centrality of shame, guilt, and moral disintegration, while also noting the construct’s deliberate exclusion from formal diagnostic systems such as the DSM-5-TR (4).

In this manuscript, moral injury is understood not as a psychiatric diagnosis, but as a clinical–moral condition that arises when individuals are unable to reconcile their actions, omissions, or survival with their internalized moral framework. Unlike post-traumatic stress disorder (PTSD), which is organized primarily around fear conditioning and threat, moral injury centers on meaning, responsibility, and trust—both in oneself and in moral authorities. This distinction is not merely theoretical: it carries direct implications for clinical stance, therapeutic goals, and the limits of symptom-focused interventions. This formulation is intentionally non-circular, emphasizing moral conflict and its consequences rather than defining the phenomenon by the moral language used to describe it.

The term moral injury captures this phenomenon: a rupture between one’s moral framework and one’s actions, or the perceived betrayal of moral expectations by others or by the system itself. Unlike PTSD, moral injury is not primarily a disorder of fear conditioning. It is an affliction of meaning, integrity, and trust. Those affected often present with pervasive guilt, shame, existential disorientation, and loss of connection with community and self-worth. Their suffering is moral and spiritual before it is psychological.

While moral injury has likely accompanied warfare throughout history, contemporary armed conflict introduces distinct conditions that intensify moral dissonance and complicate moral repair. Modern warfare is increasingly asymmetrical, technologically mediated, and conducted within blurred boundaries between combatant and civilian, defense and aggression, action and omission. Decisions with irreversible moral consequences may be made under extreme time pressure, partial information, and diffuse chains of responsibility, often at physical or psychological distance from their human impact.

These features do not create moral injury *de novo*, but they alter its phenomenology. Moral responsibility becomes fragmented, moral authority less visible, and opportunities for communal moral repair increasingly scarce. For some service members, this results in forms of moral suffering that elude existing diagnostic categories and resist conventional clinical narratives of trauma, particularly those centered exclusively on fear-based psychopathology.

Clinically, moral injury challenges the traditional psychiatric orientation toward symptom reduction and corrective intervention. While pharmacotherapy and trauma-focused treatments may alleviate comorbid PTSD or depression, they often fail to address the moral dimension of suffering at the core of moral injury. What is required is not the correction of distorted cognitions, but the acknowledgment of moral pain that may be grounded in intact values rather than pathology. This clinical orientation aligns with emerging practice-based frameworks that conceptualize moral injury as a process of moral repair rather than symptom elimination, emphasizing narrative integration, communal acknowledgment, and the restoration of moral agency (5).

In this context, humility refers not to a general therapeutic virtue, but to a specific clinical stance: the recognition that psychiatry cannot adjudicate moral innocence or guilt, nor offer absolution. The clinician’s role shifts from expert interpreter to moral witness—accompanying patients as they grapple with responsibility, loss of moral coherence, and the limits of repair. This stance marks a substantive departure from standard psychiatric models and underscores what is distinctive about treating moral injury as opposed to other forms of psychological distress.

Moral injury is not unique to military contexts, nor to psychiatry. Similar forms of moral suffering have been described among healthcare workers, humanitarian responders, journalists, and others exposed to ethically compromising situations. However, moral injury poses a particular challenge for psychiatry because it resists medicalization while presenting in clinical settings, often through the language of symptoms, diagnoses, and disability. The tension between moral suffering and psychiatric classification places clinicians in an ethically complex position that few other professions routinely inhabit.

In societies where military service is nearly universal, moral injury resonates far beyond the clinic. It shapes collective discourse on responsibility, empathy, and the moral cost of defense. For clinicians, the challenge is not only to treat individuals but to help sustain a moral community that can bear witness to their pain without recoil or denial. When a society can tolerate listening to its veterans’ moral anguish, it affirms the shared humanity that warfare threatens to erode.

From a military systems perspective, the clinical recognition of moral injury carries implications that extend beyond individual treatment encounters. Military organizations shape moral expectations through training, rules of engagement, command structures, and institutional narratives about duty, responsibility, and legitimacy. When service members experience moral injury, the source of suffering is often not only the precipitating event, but the perceived absence of acknowledgment, containment, or moral dialogue within the military system itself. This places military psychiatry at a critical interface between individual moral suffering and institutional responsibility (3, 5).

At an operational level, attention to moral injury suggests the need for earlier and more structured opportunities to process moral conflict within military settings, including peer-based forums, leadership-mediated debriefings, and clinical spaces that permit moral reflection without immediate diagnostic labeling. At a strategic level, it underscores the importance of moral literacy within military mental health services, command training, and post-deployment care, particularly in contemporary conflicts characterized by asymmetry, ambiguity, and constrained agency. Addressing moral injury within the military context is therefore not only a matter of individual care, but a component of sustaining ethical coherence, trust, and psychological resilience within the force as a whole (3, 5).

A further unresolved question concerns the moral and institutional status of moral injury within military cultures. Many armed forces have gradually come to recognize post-traumatic stress disorder as an honorable wound - an injury sustained in the course of service, often accompanied by formal acknowledgment, care, and symbolic recognition. Moral injury, however, occupies a far more ambiguous space. If PTSD is increasingly understood as a cost of exposure to threat, moral injury confronts militaries with suffering that arises from moral agency, judgment, and responsibility.

This raises difficult questions that remain largely unaddressed within military systems. Is moral injury an honorable wound of service, or does it remain implicitly stigmatized because it implicates moral choice rather than physiological or psychological reaction? How should militaries understand the suffering of service members who experience moral injury after refusing to carry out orders they perceive as illegal or profoundly immoral? And what does it mean for a military institution to acknowledge moral injury without framing it as either weakness or disobedience?

Writing from Israel - a context marked by prolonged conflict, compulsory service, and continuous ethical tension - these questions are not abstract. They shape how soldiers interpret their own suffering, how clinicians are permitted to listen, and how institutions draw the boundaries between honor, responsibility, and injury. Moral injury thus challenges not only clinical models, but military narratives of legitimacy, obedience, and sacrifice. Whether militaries are willing to engage these questions may ultimately determine whether moral injury remains a silent burden or becomes a shared moral cost of modern warfare.

The implications for military psychiatry are profound. Training should include moral literacy - an awareness of ethical complexity and emotional consequence. Debriefing and peer-support structures should enable service members to process moral conflict before it calcifies into despair. Clinical systems must recognize that guilt and shame are not merely cognitive distortions to be challenged, but expressions of conscience seeking restoration.

Clinicians themselves are not immune. Bearing witness to the moral suffering of others can elicit secondary moral distress - a quieter echo of the healer’s paradox. Acknowledging this vulnerability within our professional culture is essential to sustaining compassion and integrity.

Moral injury reminds us that psychiatry’s vocation is not only to alleviate distress but to engage with the moral dimensions of human experience. In the aftermath of modern warfare, where boundaries blur and meaning fractures, our role extends beyond diagnosis. It is to accompany those who struggle to remember what it means to remain human—and, in doing so, to preserve our own humanity as well, in a profession that is increasingly asked to contain suffering that cannot be neatly diagnosed or resolved.

## Data Availability

The original contributions presented in the study are included in the article/supplementary material. Further inquiries can be directed to the corresponding author.
